# A Review on the Feasibility and Efficacy of Home-Based Cognitive Remediation in People with Multiple Sclerosis

**DOI:** 10.3390/jcm13071916

**Published:** 2024-03-26

**Authors:** Angela Boschetti, Elisabetta Maida, Michelangelo Dini, Marta Tacchini, Giulia Gamberini, Giancarlo Comi, Letizia Leocani

**Affiliations:** 1 Experimental Neurophysiology Unit, Institute of Experimental Neurology—INSPE, IRCCS San Raffaele Scientific Institute, 20132 Milan, Italy; boschetti.angela@hsr.it (A.B.); tacchini.marta@hsr.it (M.T.); 2 San Raffaele Vita-Salute University, 20132 Milan, Italy; 3 Department of Advanced Medical and Surgical Sciences, University of Campania “Luigi Vanvitelli”, 80138 Naples, Italy; 4 Department of Neurorehabilitation Sciences, Casa di Cura Igea, 20144 Milan, Italy

**Keywords:** telerehabilitation, cognitive remediation, multiple sclerosis, feasibility, efficacy

## Abstract

Cognitive impairment affects 34–65% of People with Multiple Sclerosis (PwMS), significantly impacting their quality of life. Clinicians routinely address cognitive deficits with in-clinic neuro-behavioural interventions, but accessibility issues exist. Given these challenges, coupled with the lifelong need for continuous assistance in PwMS, researchers have underscored the advantageous role of telerehabilitation in addressing these requirements. Nonetheless, the feasibility and efficacy of home-based cognitive remediation remain to be firmly established. In this narrative review, we aimed to investigate the feasibility and efficacy of digital telerehabilitation for cognition in PwMS. Thirteen relevant studies were identified and carefully assessed. Regarding the feasibility of cognitive telerehabilitation, evidence shows adherence rates are generally good, although, surprisingly, not all studies reported measures of compliance with the cognitive training explored. Considering the efficacy of rehabilitative techniques on cognitive performance in PwMS, findings are generally inconsistent, with only one study reporting uniformly positive results. A range of methodological limitations are reported as potential factors contributing to the variable results. Future research must address these challenges, as more rigorous studies are required to draw definitive conclusions regarding the efficacy of home-based cognitive remediation in PwMS. Researchers must prioritise identifying optimal intervention approaches and exploring the long-term effects of telerehabilitation.

## 1. Introduction

Multiple Sclerosis (MS) is an inflammatory demyelinating disease of the Central Nervous System (CNS) [1]. It is caused by an autoimmune condition which largely leads to the loss of myelin in the white matter of the brain, spinal cord and optic nerves, with the resulting pathological features being diffuse and focal areas of inflammation, demyelination, gliosis, and neuronal injury [2]. MS is the most common non-traumatic cause of neurological disability in younger adults [3] and estimates suggest 2.8 million people world-wide are living with the disease. Since 2013, MS prevalence has risen. Across 75 reporting countries, the pooled incidence rate stands at 2.1 cases per 100,000 persons annually, with the average age of diagnosis being 32 years, and females being twice as likely as males to be affected by MS. [4]. Given the widespread nature of the lesions within the CNS [5], MS symptoms can be quite heterogeneous, with patients showing impairment in motor activity, sensory functions, visual functions, cognition, and behaviour. MS disease-modifying therapy aims at slowing down the progression of the disease and treating the symptoms, while rehabilitation is primarily targeted towards some degree of recovery of motor and cognitive functions [3].

Around 40–65% of individuals with MS suffer from Cognitive Impairment (CI) [6,7], with deficits manifesting during the disease, even in patients with probable MS, early MS and clinically isolated syndrome [8,9]. The cognitive functions mostly affected are attention, information-processing speed, verbal memory, visuospatial skills, and executive functions [10,11]. CI impacts social, work, and day-to-day living [12], and it is related to a lower quality of life [13,14]. Indeed, People with MS (PwMS) report lower chances for employment, a greater need for personal assistance, lower likelihood to engage in social activities [7], more difficulties in parenting [15] and greater impairment in some instrumental skills, such as driving [16,17]. Interestingly, the effects of pharmacological interventions are limited in treating cognitive symptoms in MS [18]. Roy and colleagues conducted a comprehensive review of the impact of pharmacotherapy on cognitive impairment in MS. Their analysis covered measurements, pathophysiology, and risk factors for cognitive dysfunction in MS, along with clinical trials of pharmacotherapy, including disease-modifying treatments and symptom-management therapies (SMTs). They found limited evidence from well-designed trials, with intramuscular interferon (IFN)-β1a showing significant benefits in cognitive processing speed and memory. Other treatments like IFN-β1b and natalizumab showed potential but lacked robust evidence. However, the literature on SMTs, such as acetylcholinesterase inhibitors and psychostimulants, provided mixed results, with methodological limitations hindering firm conclusions [19]. Not surprisingly, there has been a growing interest in neuro-behavioural approaches as means of managing cognitive dysfunction in individuals with MS [20]. The purpose of cognitive remediation techniques is mostly to strengthen residual capacities and promote the learning of new strategies, eventually leading to improved cognitive performance [3].

Behaviourally based cognitive remediation provides many advantages, i.e., it is non-invasive, and has no side effects typical of medications). It might have benefits such as minimising cognitive deficits, mitigating the negative influence of CI and fostering patients’ understanding of their cognitive challenges, empowering them to manage daily activities effectively [21]. Different evidence-based reviews exist looking into the efficacy of cognitive rehabilitation in MS [22,23,24,25,26] A Cochrane systematic review provided low-level evidence for neurorehabilitation reducing cognitive symptoms in PwMS. Specifically, cognitive training was observed to enhance memory span and working memory. While cognitive training did exhibit significant effects on specific subcategories of cognitive performance, most comparisons did not produce statistically significant results. When paired with other neuropsychological rehabilitation techniques, cognitive training demonstrated improvements in attention, immediate verbal memory, and delayed memory, but not in information-processing speed, memory span, working memory, immediate visual memory, executive functions, visual functions, or verbal functions [21]. While contrasting findings are not uncommon in relation to the efficacy of cognitive rehabilitation in MS [22,23,24,25,26], a recent narrative review affirmed that despite methodological limitations in certain studies and considerable diversity in protocols, the overall findings tend to support the efficacy of cognitive rehabilitation, suggesting it is a promising approach [25].

Conventionally, cognitive rehabilitation requires the patient to travel to the clinic for repeated one-to-one sessions with the clinician for a set period of time, which may last several weeks. This may be a costly approach and not entirely feasible for some patients with MS [27,28]. Chiu and colleagues examined the specific barriers to accessing healthcare services in PwMS and found geographical location and transportation to be a frequent issue [29]. In summary, reported concerns in MS patients included the following: (a) living in remote and/or rural regions [30]; (b) suffering from fatigue, which may increase the burden of travel [30,31]; (c) inefficiency of existent transit services [31,32,33,34,35]; (d) needing to depend on family members or friends for assistance, and thus having to also rely on others’ availability when scheduling appointments; and (e) having to make appointments during working hours [30,31]. Home-based options for cognitive rehabilitation may offer a fundamental tool to overcome some of these issues while potentially reducing healthcare costs by limiting in-person visits [36].

Naturally, telerehabilitation has received much attention in the context of the COVID-19 pandemic, for which many healthcare services were limited to emergency care to reduce the risk of contagion and due to lockdowns in many countries [37]. This rendered even more evident the need for support services which could allow for the continuity of healthcare even when external circumstances may impede in-person medical assistance. Moreover, since the management of symptoms and impairment in MS often requires a comprehensive set of continuous treatments to promote patients’ well-being during their lifespan [29], it may be valuable to have the possibility to extend medical care to home-based services during the disease course, particularly in the context of prolonged treatment and monitoring of outpatients. To this purpose, home-based care was observed to offer significant advantages. These included a notable reduction of 65% in missed workdays among patients [38], a particularly crucial benefit given the prevalence of MS among young adults who are often in their prime working years. Additionally, home-based care resulted in an average saving of 258 km in travel distance and a 17% reduction in lodging costs [38]. Regarding the acceptability of such tools, researchers suggest an interest among PwMS in utilising telecommunication technologies for rehabilitation services. Remy and colleagues conducted a cross-sectional survey within an outpatient neurological facility to investigate the accessibility of telecommunication technologies and rehabilitation services among PwMS, along with their willingness to utilise such technologies for physical rehabilitation purposes. A total of 200 individuals with MS responded. Over half of the respondents expressed interest in receiving exercise programs via telehealth platforms, particularly through internet-based platforms and mobile applications. The results, thus, indicated a predisposition among PwMS for utilising telecommunication technologies for rehabilitation, with a majority having access to the necessary technology. Furthermore, patients with mild disability and those engaged in professional activities demonstrated a higher inclination towards telerehabilitation [39]. Moreover, even if results on home-based telerehabilitation for MS may not always be consistent, several studies found interventions to be feasible and effective [40,41]. Patient and provider satisfaction with telehealth across various specialties has been high, suggesting home-based digital rehabilitation holds promise in addressing accessibility barriers for MS patients [42]. Specific to cognitive remediation, users have found telerehabilitation to be acceptable [43] and feasible [44].

Owing to the continuous technological advancement, new forms of technology-based programmes have simultaneously gained interest as potential tools for rehabilitation in PwMS. Examples of such techniques are robotic training, computerised serious games, virtual reality systems and video games. Technology-based rehabilitation provides many advantages: (a) tasks can be built to closely resemble activities of daily living; (b) frequent repetitive training can be easily implemented; (c) multisensory feedback can be available; (d) training difficulty can be adapted to patient’s ongoing performance; and (e) training can provide an engaging and motivating environment [45,46]. There is compelling evidence indicating that the latest advancements in rehabilitation technologies have yielded significant benefits for individuals with MS. For instance, Leocani and colleagues have proposed that virtual reality systems hold potential for identifying impairment in PwMS [47]. Additionally, a randomised controlled trial employing a serious games platform not only garnered positive feedback in terms of user experience and motivation, but also resulted in clinically meaningful enhancements [48]. Similarly, a systematic review highlighted the efficacy of clinical applications and robotic-assisted training in facilitating functional recovery [49]. Moreover, it was shown that when virtual reality was integrated with conventional training, there was a potential acceleration in recovery and rehabilitation [50] and rehabilitation through computer-based software not only enhanced cognitive functions immediately post-training, but also maintained some of these improvements during follow-up appointments [51]. Also, many technology-based tools allow for home-based asynchronous rehabilitation, meaning patients can complete the training at home at any time [52]. This may be particularly advantageous in PwMS who struggle to schedule their appointments due to work and other commitments.

Given the growing interest in telerehabilitative techniques based on the newest technologies, it remains to be established whether there is solid evidence regarding their efficacy in alleviating cognitive deficits in MS, and whether they can be a feasible option in these patients. The aim of the current narrative review was to present and critically evaluate recent research findings uniquely about home-based digital cognitive rehabilitation in MS in order to explore its feasibility and efficacy. This includes software, apps, web-based platforms, virtual reality applications or any digital tool designed and/or applied to train cognitive domains. Interventions targeting both domain-specific cognition and a broad spectrum of cognitive domains were equally considered, without excluding any specific cognitive area.

## 2. Materials and Methods

A comprehensive literature search was conducted using the online database PubMed to gather relevant articles and information for this narrative review. The search was performed between May 2023 and September 2023, utilising the following keywords: “home-based rehabilitation” or “remote rehabilitation” or “telerehabilitation” and “cognition” or “cognitive rehabilitation” or “cognitive remediation” or “cognitive training” and “Multiple Sclerosis”. Exact strings utilised combinations of the provided keywords, for instance “telerehabilitation and cognition and Multiple Sclerosis”. The only filter applied to the search was a customised publication date range to include articles published between January 2005 and September 2023. This timeframe was chosen to encompass recent literature while ensuring a sufficiently broad scope. The search strategy aimed to identify studies focusing on home-based digital cognitive rehabilitation interventions in PwMS. The inclusion criteria were specified to include only studies which involved an intervention, with detailed empirical outcomes, which strictly investigated the utilisation of home-based digital rehabilitation for cognition in PwMS, thus including studies which looked at cognitive rehabilitation at home through the use of digital tools such as computer programmes, software, web-based platforms, apps, virtual reality systems, video games, serious games or any other specific digital tool. Studies could be clinical trials, randomised controlled trials, but also non-randomised controlled trials. Non-controlled studies were included alongside controlled trials for this narrative review to enrich the discussion and provide a more nuanced understanding of the topic. Both domain-specific cognitive interventions and those targeting a broad spectrum of cognitive domains were given equal consideration, without any exclusions based on specific cognitive areas. The studies were required to be written in English to ensure accessibility and comprehension for the intended audience. The studies had to involve a sample of adult MS patients and any research involving other medical conditions or MS paediatric population was not considered. Narrative or systematic reviews, and theoretical articles were not of interest, nor were studies looking at rehabilitation at home paired with “in-person” sessions or telerehabilitation paired with at-home neurostimulation. Narrative or systematic reviews were excluded because the aim of this review was to provide a detailed analysis of primary research articles. Studies examining rehabilitation paired with in-person sessions or neurostimulation were excluded to maintain the focus on home-based digital cognitive rehabilitation interventions. Including studies with additional components such as in-person sessions or neurostimulation could introduce confounding variables that would complicate the investigation of the efficacy and feasibility of home-based digital interventions specifically. By excluding these studies, we aimed to provide a more focused and streamlined review. After retrieving the initial set of articles from the literature search and reference section, the titles and abstracts were screened to narrow down the search for relevant studies according to the predefined eligibility criteria.

The screening process was conducted independently by two reviewers to minimise bias, and disagreements would be eventually resolved through discussion. A total of 22 articles were identified as potential research studies focusing on telerehabilitation interventions in PwMS. Upon closer examination of the full articles, it was determined that 9 out of the initial 22 identified articles either did not meet the aforementioned inclusion criteria for this review or were excluded based on the established exclusion criteria. Thus, 13 relevant studies meeting the inclusion criteria were suited to be included in the review. In this narrative review, the primary focus was on synthesising and discussing the existing evidence surrounding home-based digital cognitive rehabilitation interventions in MS patients. Given the narrative nature, the intention was to comprehensively cover relevant research without conducting a formal evaluation of study quality. This approach allowed us to include a broad range of studies and discuss their findings in a narrative manner. While we did not explicitly perform a formal quality assessment, we recognise the importance of factors such as sample size, randomisation, blinding, controls, and potential bias in interpreting research outcomes. These considerations were set to be discussed in the narrative to provide context and interpretation of the findings. Data synthesis from the included studies involved a qualitative overview of the studies; a quantitative report on the intensity of the interventions reported by each study, specifying duration of one single intervention session, number of sessions of intervention per week, total number of weeks of interventions and total hours of intervention; a quantitative report of feasibility of telerehabilitation evaluated based on adherence to intervention, depending on the metric used by each article; and a quantitative report of efficacy of interventions, evaluated by looking at pre–post mean differences as reported by each study.

## 3. Results

### 3.1. An Overview of the Studies on Digital Telerehabilitation

Thirteen studies investigating the use of computer-based software or programmes, applications, or video games for cognitive rehabilitation at home (digital telerehabilitation) were found and selected to be included in the current narrative review. The studies collected and reported below were published between the year 2007 and the year 2021. Table 1 provides an overview of the studies, while Table 2 provides more details regarding sample characteristics.

Table 1 presents an overview of the included studies on digital telerehabilitation. Each entry includes the first author’s surname and year of publication and the groups’ size differentiating between intervention and control group. The study objectives, targeted cognitive domains, outcome measures, and the presence of follow-up assessments are also detailed. Moreover, the blinding of the study, whether participants were randomly allocated to intervention or control group and whether an active control condition was implemented is specified. The entries are organised from the earliest to the most recent study for easy reference.

Table 2 presents an overview of the characteristics of the samples of the included studies on digital telerehabilitation. Each entry includes the first author’s surname and year of publication, disease type of participants, mean age, gender, mean education, mean Expanded Disability Status Scale (EDSS), and whether any dropouts were reported. The entries are organised from the earliest to the most recent study for easy reference.

Twelve studies involved predominantly patients with Relapsing Remitting MS (RRMS), while seven were also able to include patients with Secondary Progressive MS (SPMS), and only three involved patients with Primary Progressive MS (PPMS). This is not surprising considering that RRMS is the most common form of the disease [64]. The majority of the research papers looked at the efficacy of home-based cognitive rehabilitation programmes in alleviating cognitive deficits (10), while a few had the goal of establishing the feasibility of telerehabilitation (2). Only one study investigated two different training schedules, still looking at the efficacy of each type of training on cognition.

Two studies focused exclusively on the rehabilitation of working memory [54,59]. Vogt and colleagues used the computerised working memory training programme BrainStim [65], comparing two different training schedules (high intensity vs. low intensity) with no training. The programme involved three modules aimed at the remediation of spatial orientation, visual memory for objects and memory for numbers, and it was adaptive to the ongoing performance of participants [54]. Pedullà and colleagues opted for working memory training with the COGNI-track app, comparing adaptive vs. non-adaptive training. Patients in the adaptive training group performed exercises with increasing-decreasing levels of difficulty based on their performance, while patients in the non-adaptive training group performed exercises at a constant low-level of difficulty. Exercises included a visuospatial working memory task, an “operation” N-back task and a “dual” N-back task [59].

A multicentre study focused on the rehabilitation of attention using the computerised Attention Processing Training (APT) programme, targeting different components of attention. For this purpose, Amato and colleagues recruited PwMS who showed impairment on at least two out of seven attention tests. Interestingly, the training exercises were organised in a hierarchical manner to train different components of attention. To provide an example of such training, the participants were asked to identify target numbers or letters in the presence of distractor images and noises. An active control group undergoing sham computer training (reading and comprehension, description of pictures, etc.) was included for comparison [6].

The study by Shatil and colleagues opted for personalised cognitive training, which was based on the difficulties emerging from the neuropsychological examination conducted with the computer programme CogniFit Personal Coach (N-CPC). Essentially, the tasks used for cognitive training were determined by individual performance on the N-CPC so that each person would have a specific individualised training regime. The participants were then allocated to intervention or control groups. Training was conducted with the same computerised training programme CogniFit Personal Coach used for the neuropsychological evaluation. [55].

Lastly, nine studies investigated multi-domain cognitive rehabilitation [46,53,56,57,58,60,61,62,63]. Hildebrandt and colleagues implemented a home-based software for rehabilitation. Specifically, they utilised a Compact Disk with memory and working memory tasks. Patients in the training group had to memorise a list of words (within a semantic category) to be recalled after they had been distracted with a series of calculations. The software was adaptive and provided acoustic and visual feedback [53]. Charvet and colleagues conducted a pilot study in which they used the adaptive training web platform Lumosity (Lumos Labs, Inc. Lumosity. Lumos Labs, 2015) to train the most common areas of cognition affected in MS, including information-processing speed and working memory. The control group was asked to complete sham activities through a commercially available programme called Hoyle puzzles and board games [46]. De Giglio and colleagues, in a pilot study, tried to rehabilitate MS individuals with the videogame Dr Kawashima’s Brain Training (DKBT, Nintendo, Kyoto, Japan) and a video game console, particularly aiming at the training of executive functions, working memory and processing speed. The training was made up of different games with specific goals. For example, the game calculation would have participants solve mathematical problems as fast as possible [56]. Hancock and colleagues collected pilot data on computerised cognitive training with the programmes Posit Science InSight and Brain Twister visual n-back. Training focused on processing speed and working memory with tasks that resembled games. The control group also received the same training but with a constant low level of difficulty, while for the intervention group, the training was continuously changing to be more and more challenging [57]. Campbell and colleagues looked at the efficacy of training through the computer software RehaCom. Training was adaptive and included modules aimed at the remediation of working memory, visuospatial memory and divided attention. Participants in the control group had to watch a series of natural history documentaries on DVDs [58]. Messinis and colleagues in a multicentre trial also used the computer-based software RehaCom. The training was adaptive and focused on attention, memory and executive functions. The control group took part in specific computer-based sham activities, such as reading and comprehension, shopping games, etc. [61]. Charvet and colleagues looked at the adaptive computer-based training programme Brain HQ developed by posit Science Corporation. The training targeted processing speed and information processing, attention, working memory and executive functions. Training was multimodal, involving the use of both visual and auditory domains. The active control group used the software Hoyle Puzzle and Board Games (2008 version) to conduct a series of sham activities [60]. Vilou and colleagues, in an exploratory study, also used the Brain HQ website developed by Posit Science to train episodic memory, attention and processing speed. Every two weeks, the exercises were reviewed by an experimenter to adjust the level of difficulty of each task based on performance. There was no intervention described for the control condition [62]. Finally, Blair and colleagues, in a pilot study, implemented the Cogmed Working Memory Training (CWMT) to mostly train working memory (both visuospatial working memory and verbal working memory) and attention. Training was adaptive. Each participant was assigned to a trained coach who would revise the participants’ progress weekly [63].

### 3.2. Training Frequency and Intensity of Digital Telerehabilitation

**Figure 1:**
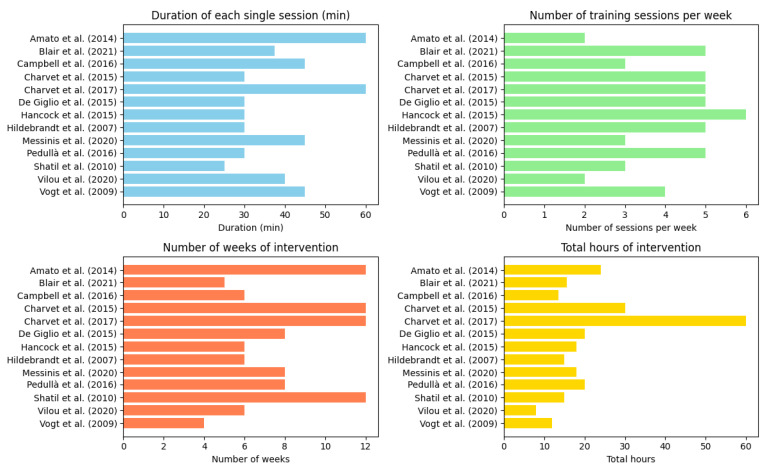
Intensity of digital telerehabilitation [6,46,53,54,55,56,57,58,59,60,61,62,63].

Overall, the studies are somewhat consistent regarding the duration of a single session, with most opting for a training session of 30–45 min (11), while only two studies implemented one-hour-long sessions. Regarding the number of sessions per week, they ranged from 2 to 6. Looking at the total number of weeks of training, there was some variability, going from a minimum of 4 weeks to a maximum of 12 weeks of training. Finally, regarding the total hours of intervention, there was even more variability, with the lowest number being 8 h of training in total, and the highest number being 60 h of training (see Figure 1 for details).

Figure 1 illustrates the training intensity across the 13 studies. All charts use horizontal bars to represent the different studies, and the length of each bar corresponds to the value of the variable for that study. The studies are ordered alphabetically for ease of reference.

### 3.3. Feasibility of Digital Telerehabilitation

Not all studies reported the feasibility of their target intervention; however, those that did found good compliance, with some variability (range = 52.1–93.75%), which could be attributed in part to differences across studies in the metric used to define compliance, as detailed below. Shatil and colleagues explored unprompted compliance and found 57.6% of participants completed at least half of the prescribed sessions [40]. Charvet and colleagues defined compliance as the adherence to over 50% of target sessions and found 80% overall compliance [46]. De Giglio and colleagues defined compliance as the ratio between the number of days in which participants performed the intervention and the total planned days, and they found 96% compliance rates [56]. Hancock and colleagues found that 93.75% of patients completed at least 80% of prescribed sessions [57]. Campbell and colleagues measured adherence as the completion of at least 75% of target training sessions, and they found 88.90% of participants satisfying the goal adherence rate [58]. Pedullà and colleagues calculated adherence as the percentage of completed training sessions out of the total percentage of expected training sessions (100%) and found 87% overall adherence [59]. Charvet and colleagues measured compliance in two manners: achievement of at least 50% of target hours of programme use or having at least completed 50% of target weeks of intervention where there was at least 50% of compliance in a week. They then reported that 58,11% of PwMS in the intervention group completed at least 50% of target sessions [60]. Finally, Vilou and colleagues reported that about half of their participants (52.1%) were compliant with the study protocol, although they did not specify the metric used to evaluate compliance [62]. Five studies failed to report a measure of adherence in their result section [6,53,54,61,63]. In their discussion, Amato and colleagues reported that participants achieved high rates of compliance based on self-reports [6]. Vogt and colleagues also reported in the discussion section that almost 100% of participants finished all prescribed training sessions [54]. Finally, Messinis and colleagues reported, once more in the discussion, that high compliance rates may be inferred by the fact that no dropouts were registered in either the intervention or control groups [61].

### 3.4. Efficacy of Digital Telerehabilitation

Findings regarding the efficacy of telerehabilitation on ameliorating cognitive symptoms in MS are variable and dependent on the study in question. Overall, the results seem to suggest there is inconclusive evidence regarding the efficacy of home-based cognitive rehabilitation on neuropsychological measures, motor measures and Patient Reported Outcome Measures (PROMs). In general, 84.62% of the studies found improvements after home-based digital rehabilitation in at least one cognitive outcome, while 66.67% found improvements in at least two cognitive outcomes. A total of 38.46% of studies found improvements in three or more cognitive outcomes explored. Interestingly, only one recent study (7.69%) reported solely positive findings following an 8-week intervention with the software RehaCom in people with SPMS [61]. In this study, participants improved after training on all outcome measures compared to the control group. Replication will be key to make more definite conclusions. All the other studies (12) reported both positive and negative results (see Table 3 for details). Of all examined studies, only four performed long-term follow-ups, and only two of them demonstrated a long-lasting effect on cognitive functions [6,59].

Table 3 shows the efficacy of digital training for each study. The table shows positive results on neuropsychological (NPS) measures in terms of improved performance after the training intervention compared to baseline. Improvements in performance from pre- to post-intervention on measures other than NPS, such as motor tests or PROMs, are also shown. Whether improvements were also seen in the control group is specified in brackets. Negative results on NPS measures and other measures are also reported. The table also shows eventual lasting performance improvements at follow-up assessment. Effect sizes for positive results on NPS measures are also displayed. The entries are organised from the earliest to the most recent study for easy reference.

## 4. Discussion

The purpose of this narrative review was to explore the feasibility and efficacy of digital cognitive telerehabilitation in PwMS. Thirteen studies were presented, offering an understanding of the subject of interest. Regarding the feasibility of cognitive telerehabilitation, researchers commonly indicate adequate rates of adherence to at-home rehabilitation protocols [46,55,56,57,58,59,60,62]. However, it is worth noting that adherence metrics may vary, with most studies primarily providing the proportion of participants who adhered to a certain percentage of scheduled training [55,57,58,60,62], lacking more detailed insights into the specific duration (minutes/hours) of completed training in comparison to the intended training duration. Only one study reported the number of days in which participants performed the intervention [56] and only one study reported the percentage of completed training vs. the percentage of total training [59]. Five studies failed to report a measure of adherence in their result section [6,53,54,61,63]. Telerehabilitation interventions may depend heavily on individuals’ adherence to the prescribed treatment; thus, for instance, only displaying the dropout rate of participants [61] may not be sufficient. Given that many factors can influence compliance, such as the degree of satisfaction with the tele-protocol [66], vacation, technological issues, health issues, occupational issues, etc. [46], researchers should monitor session attendance, the completion of assigned tasks and actual use of the provided digital tools. Improvements could also be made in reporting the number of participants who successfully complete the training, and further research is warranted to determine the threshold of completed training that signifies satisfactory compliance with the study procedures. Some existent software (e.g., RehaCom) is able to provide accurate measures of treatment adherence, offering the actual minutes of completed training vs. total minutes of programmed training for each session. Such tools should be exploited to gain more accurate insights into the feasibility of home-based interventions. Moreover, assessing reasons for non-adherence or dropouts, if possible, may also provide valuable information regarding the practicality and acceptability of telerehabilitation in real-world settings. Surely, it is crucial to report objective measures of adherence to minimise reliance solely on self-reports provided by patients and caregivers, as these have been reported to overestimate adherence in the context of pharmacological treatment [67] and at-home exercise therapy [68].

In addition, further exploration into the acceptability of telerehabilitation interventions is needed. Understanding factors such as satisfaction with telemedicine, technical issues related to the use of digital equipment remotely, influence of individual health or occupational constraints can provide valuable insights into the practicality and acceptability of such interventions in real-world settings. A recent study looking into patients’ acceptance of telemedicine in a rehabilitation setting found that obstacles to telehealth encompassed a deficiency in intrinsic motivation and perceiving communication with healthcare professionals via portals as ‘impersonal’ [69]. Moreover, a recent systematic review aimed to identify theoretical predictors influencing the acceptance of telemedicine and found that factors such as perceived usefulness of a technology and individual need for social support significantly impact end-user acceptance [70]. This implies that forthcoming studies should not solely focus on examining the feasibility of a treatment intervention but also consider its level of acceptance. Self-reported questionnaires like the System Usability Scale [71] could be considered for this purpose. In addition, assessing barriers to access, including poor e-literacy, limited internet and technology access, geographical limitations, will be essential for identifying potential challenges in implementing telerehabilitation programs effectively. For instance, people living in rural or underserved areas have been found to not have access to high-speed internet or may lack the necessary technological infrastructure to engage in telerehabilitation sessions [72]. If telerehabilitation is considered a potential solution for alleviating transportation barriers and enhancing access to healthcare services among rural residents, it remains essential to thoroughly evaluate its accessibility within this specific context. Socioeconomic factors will also deserve consideration, as they may play a role in accessing digital tools. For instance, it was seen that a number of barriers hinder the widespread adoption of telemedicine in developing countries [73]. Thus, while investigating feasibility, it will be fundamental to also assess its acceptability in real-world settings and identify any potential barriers that may hinder participation in telerehabilitation programs in order to develop strategies to address these challenges. Improving accessibility will not only enhance the feasibility of telerehabilitation interventions but also ensure equal access care opportunities.

Regarding the efficacy of the different rehabilitative techniques in improving cognitive performance in MS, there is more inconsistency in the findings. Variable results may be attributed, to some extent, to a wide range of methodological shortcomings across different studies. For example, many studies lacked double-blinding or failed to report whether the study was double-blind, single-blind or open-label [53,54,55,56,58,59,62,63]. A lack of double-blinding remains an issue as it may lead to increased cognitive bias and unreliability of findings [74]. A lack of double-blinding is often a direct consequence of inadequate control groups. This is actually the case for various studies which did not involve active sham conditions and/or adopted wait-list control groups [53,54,55,56,62,63]. This is a concern, as a lack of blinding may lead to an effect for the treatment group which is not a true effect per se but arises from different group expectations [75]. A lack of controlled randomisation, which is the gold standard in science [76], is also an issue, as a few studies failed to implement controlled randomisation [53,54,55]. Furthermore, in some instances, even when randomisation was conducted, a few studies failed to report the specific details of how this process was carried out [46,59,63]. Moreover, most studies lacked a follow-up assessment, rendering it impossible to know whether any eventual benefits of training were maintained over time [46,53,54,55,56,57,60,61,62]. This aspect is key since the lack of prolonged efficacy would likely suggest the need for longer and/or more intensive rehabilitation protocols. The frequent absence of long-term follow-ups may stem from the limited availability of patients to attend multiple repeated assessments, leading to expectations of significant losses to follow-up, which critically affect statistical power in small sample sizes such as those typically observed in studies on cognitive telerehabilitation. Therefore, new studies with longer follow-up durations are essential to reliably determine whether cognitive telerehabilitation can produce lasting benefits. This aspect should be carefully considered when performing sample size calculations, thus accounting for the power loss due to loss of patients to follow-up, in order to produce reliable data. On a positive note, a few studies tried to control for practice effects using alternate forms of neuropsychological testing, when possible [6,46,56,57], and adopting counterbalancing when administering the neuropsychological assessment [57]. Notably, one of these studies also observed long-lasting benefits in the control group, suggesting that either the sham intervention had produced a tangible improvement, or that practice effects may have affected the reliability of repeated assessments even when using alternate test versions. Lastly, it should be noted, however, that some studies have demonstrated how an alternate test version may not always be strictly equivalent [77,78]. Therefore, guidelines for the use of alternate test versions should be established in order to further reduce heterogeneity across studies. Interestingly, one study tried to control for practice effects by subtracting the difference in mean scores at the neuropsychological tests in the control group (no active sham condition) from the difference in mean scores in the intervention group [62]. Furthermore, a few studies [46,53,59,60] also reported an intent-to-treat analysis which may be useful to help preserve randomisation, carry out a realistic evaluation of an intervention, and minimise the risk of biases due to non-compliance and dropouts [79]. Two studies also provided a Reliable Change Index (RCI) analysis [57,62], which is optimal as RCI is a statistical measure that helps determine whether there was a significant change in score at the individual score level on a particular assessment test. This is useful to determine whether the observed effect is actually a real change or whether it is due to random variability or measurement error [80].

As an additional limitation, studies on cognitive telerehabilitation in MS tend to have relatively small samples, which may reduce statistical power, robustness, and the generalisability of the results [81]. Furthermore, there is the issue of heterogeneity among PwMS, as they may exhibit distinct cognitive profiles depending on the location of lesions within the CNS [10]. Consequently, grouping patients together based on diagnostic labels could potentially overlook the variations in cognitive symptomatology. This challenge in MS research urges researchers to devise ideal strategies to effectively account for disease heterogeneity. Undoubtedly, controlling for the baseline characteristics and cognitive profiles of participants is of the utmost importance. A common approach is to ensure that the intervention and control groups have similar baseline characteristics through stratified controlled randomisation. If any discrepancies arise between the two groups, appropriate adjustment analysis should be employed to account for these differences [82]. Of greater significance, future studies should strive to overcome recruitment challenges by aiming to increase sample sizes. Multicentre studies will be key in this respect. This expansion would enable grouping analysis, which can aid in identifying disease subtypes based on baseline characteristics [83]. Indeed, a multicentric study on the effectiveness of cognitive rehabilitation and exercise in the clinic (N = 284) was recently published [84]. Similar efforts should be encouraged in the framework of telerehabilitation, and indeed one could argue that telerehabilitation may afford a higher degree of standardisation across centres, thus facilitating multicentric and decentralised trials. Ultimately, such identification may facilitate predictions regarding which group of patients may be more likely to respond positively to a certain treatment. Similar work has been carried out to explore whether medications are more effective in some MS patients compared to others [85]. Moreover, cluster analysis can provide support in this context, as it provides a data-driven approach to classify patients into homogeneous groups based on specific characteristics, allowing distinct patterns to be identified. This approach facilitates individualised adaptation of cognitive rehabilitation interventions to meet individual needs. While cluster analysis has already been successfully applied in some neurological disorders within the context of cognitive rehabilitation [86,87], there is still room for significant advancement in the field of MS and in the application of digital technologies. The possibility to extend this methodology to the cognitive field may help shed more light on the effectiveness of cognitive rehabilitation. In this regard, a multicentre cross-sectional study applied latent profile analysis to cognitive tests to identify cognitive phenotypes (N = 1212). Cognitive phenotypes can represent a more meaningful measure of the cognitive status of PwMS and can help tailor cognitive rehabilitation strategies [88]. Another approach, instead, may be personalised rehabilitative intervention based on individual deficits emerging from the neuropsychological assessment. By identifying and targeting specific deficits that a person has, rehabilitation efforts can be tailored to their unique needs, hypothetically maximising the potential for improvement. One of the studies included in this review looked at personalised treatment regimens with the use of an app and found some improvements with moderate to large effects [55]. More research will be needed to also explore the possibility of customised treatment.

The variability in findings within the field of telerehabilitative methods could also be partly attributed to the heterogeneity of these techniques as well as the heterogeneity of inclusion criteria employed in studies. For instance, some studies recruited participants based on self-reported cognitive deficits [46,54,57] rather than objectively measured cognitive impairment using neuropsychological tests, or individuals with intact cognitive performance [56]. While this approach may be driven by the primary focus on assessing the feasibility of a specific protocol, it becomes crucial, particularly when presenting the efficacy of a telerehabilitation intervention, to recognise that individuals with cognitive impairment could potentially respond distinctively compared to those with intact cognitive abilities. Interestingly, when Hancock and colleagues specifically examined subjects with cognitive impairment on the Symbol-Digit Modalities Test (SDMT), excluding cognitively intact individuals, the treatment effect became statistically non-significant [57]. This may suggest that cognitively impaired individuals may respond less to cognitive training compared to those who are cognitively intact, possibly due to lower brain reserve and/or cognitive reserve. In this regard, however, there are conflicting results. The study performed by Whitlock and colleagues on a sample of 39 older adults aged 60–77 suggests that older adults with lower cognitive functioning may stand to benefit more from cognitive training [89]. Nevertheless, not screening for cognitive impairment may also pose an ethical concern as it may result in ‘treating’ cognitively intact participants who might still benefit from cognitive boosting interventions but with limited clinical impact. Emphasising the prioritisation of enhancing clinical relevance in research is crucial, considering the importance of generalising and translating findings to real-world situations. Indeed, it is crucial to examine whether study results have a substantial impact that holds significance for patients in their daily lives. While self-report questionnaires can serve as an initial measurement of perceived improvements in functioning, exploring methods to objectively assess meaningful functional changes is an intriguing avenue to explore.

The selection of appropriate outcome measures can also be challenging. A measure should be sensitive to the cognitive domain of interest and relevant for the intervention being evaluated. For instance, Campbell and colleagues used the subtests from the Brief International Cognitive Assessment for MS (BICAMS) to measure cognitive improvement after rehabilitation with RehaCom software modules targeting working memory, visuospatial memory and divided attention [58]. BICAMS subtests are the SDMT (information-processing speed and sustained attention), the California Verbal Learning Test-II (CVLT-II; verbal learning and memory) and the Brief Visuospatial Memory Test Revised (BVMT-R; visuospatial learning and memory) [90]. Although working memory refers to a set of cognitive systems that are considered essential for retaining and manipulating information while engaging in complex tasks such as reasoning, comprehension, and learning [91], and thus is likely involved in various neuropsychological tests, it is usually measured with tests that require the active maintenance and manipulation of information [92]. As a result, perhaps, it would have been interesting to also include measures more sensitive than BICAMS to a possible improvement in working memory following rehabilitation. Similarly, for instance, it would have also been interesting to include a verbal memory module as cognitive training. Indeed, no significant improvement in BICAMS test CVLT-II was reported after cognitive training. Since no verbal memory training was involved in the intervention, this finding is perhaps not surprising. Interestingly, the authors showed that there was a functional imaging difference between the control and intervention groups, with the intervention group showing more activation in the prefrontal cortex and right temporoparietal regions in response to working memory tasks (in functional Magnetic Resonance Imaging—fMRI), which, however, was not reflected in the scores obtained by the BICAMS tests. Again, this is suggestive that BICAMS neuropsychological subtests may not be sensitive enough to working memory changes [58]. This particular case serves as an illustrative example highlighting the challenge of selecting relevant outcome measures. When evaluating the cognitive gains resulting from an intervention, it becomes crucial to employ sensitive measures of change. Without such appropriate measures, the interpretation of cognitive improvements can be challenging. Some researchers, for instance, also reported a composite score of cognition derived from an average of the different neuropsychological tests used [46,60]. Combining multiple measures into a composite score can provide a more comprehensive assessment of cognitive function and it may increase the statistical power to detect treatment effects when interventions are designed to target multiple cognitive domains. Conversely, this approach could lead to a loss of statistical power when assessing the efficacy of domain-specific interventions. Therefore, it remains important to carefully select outcome measures and apply appropriate statistical analyses to create and interpret composite scores.

A further challenge when designing telerehabilitation experiments lies in the decision of treatment intensity and duration. Only one study included in the review compared different training schedules, with no significant differences in pre–post intervention scores at neuropsychological testing, concluding that the effects of training were independent of training intensity. The only difference found was in the CORSI block backwards, for which there was an improvement only in the distributed training group [54]. A meta-analysis examining the effects of cognitive intervention in individuals with Mild Cognitive Impairment (MCI) found that the number of sessions does not significantly impact the training effect. Contrary to expectations, studies with longer session durations or training durations did not yield larger effect sizes [93]. Supporting this, a recent study with a large sample size indicated a minimal association between the number of training sessions and the observed transferable benefit [94]. Upon qualitative and visual examination of the relationship between training outcomes and the total duration of training in the present study, it seems that longer interventions do not necessarily yield greater effectiveness compared to shorter ones. However, it is important to note that this observation is solely based on a visual inspection of the data. It remains uncertain what the optimal intensity and duration of training might be. [58]. Whether it is a possibility that, in some research scenarios, the duration of the intervention may have been too short to allow for significant results to emerge in the cognitive measures under investigation [93], the possible negative impact of increasing duration and/or frequency of telerehabilitation sessions on adherence should also be carefully evaluated. Perhaps, given the above considerations and mostly favourable feasibility results, future cognitive telerehabilitation protocols may benefit from increasing treatment duration (e.g., 12 weeks) while avoiding increasing frequency. Certainly, further investigations are required to provide clearer insights into the ideal schedule of training for optimal efficacy.

Another significant challenge is the choice of cognitive functions/domains to target for telerehabilitation. Several approaches could be implemented in this regard, as demonstrated by the available studies so far. Some studies focused on executive functions, attention and working memory, which are known key contributors of performance across a wide range of neuropsychological tests [95,96]. Others targeted the domains which are most commonly affected in PwMS (processing speed, verbal and visuospatial memory). Only one study designed a telerehabilitation protocol specifically targeting the cognitive functions which were found to be impaired on a patient-by-patient basis [55]. Each approach has its pros and cons. Specifically, the more individualised a treatment plan is, the harder it becomes to standardise treatment and outcome measures, and to generalise the results. Moreover, this approach is contingent on the availability of comprehensive neuropsychological baseline assessments, which could be infeasible in everyday clinical practice. More generalised approaches, on the other hand, could be more easily implemented and standardised, but they may lack sensitivity when the outcome measures are not specific for the cognitive functions/domains which are affected in the clinical sample. Targeting the most commonly affected cognitive domains in PwMS (processing speed, verbal and visuospatial memory) may represent an adequate compromise since it allows researchers to reach a higher degree of standardisation while simultaneously providing specific treatment protocols. Finally, this would enable researchers to select gold-standard outcome measures (i.e., the BICAMS) which are sensitive to changes in the cognitive domains being treated.

Despite the heterogeneity of results and the highlighted criticisms, cognitive telerehabilitation has the potential to be a promising option for the management of PwMS, with positive adherence rates. However, methodological challenges affect efficacy interpretation, long-term follow-ups are lacking, and disease heterogeneity complicates findings. Cognitive telerehabilitation offers PwMS the possibility to manage their rehabilitation independently in a familiar environment, thus expanding access to services, especially for those patients who have difficulty reaching traditional in-person centres. This may significantly improve patients’ quality of life by allowing them to participate in cognitive rehabilitation interventions without the spatial and temporal limitations of traditional programs. Additionally, this approach may reduce the burden on caregivers, enabling them to actively participate in the intervention process without physically attending appointments. Finally, the reduction in costs associated with remote rehabilitation could improve accessibility and alleviate financial burdens on patients, their families, and the national healthcare system.

In conclusion, it is imperative to address the methodological concerns discussed above to uphold the validity, reliability, and generalisability of research findings related to cognitive telerehabilitation in MS. Most notably, it is essential to (a) pursue the standardisation of intervention protocols in cognitive telerehabilitation to minimise study variability and facilitate the comparison of results; (b) favour the adoption of double-blind randomised controlled trials to achieve a high degree of reliability in assessments; (c) encourage the use of objective and correct cognitive screening measures such as those recommended by international consensus statements (e.g., the BICAMS) while also paying close attention to include appropriate tests for the cognitive functions targeted by the rehabilitation protocol; (d) broaden and stratify the study sample by promoting multicentre studies to ensure a greater representativeness of the study population; (e) account for expected dropout rates when performing sample size calculations; and (f) implement long-term follow-up to adequately evaluate the effectiveness of the intervention over time, carefully considering the use of alternate test forms to reduce bias from practice effects of repeated testing. The adoption of these proposed guidelines will be able to contribute significantly to the improvement in quality of scientific research in the context of cognitive telerehabilitation. Furthermore, ongoing and relentless technological research will enable the development of ever more cutting-edge digital solutions for cognitive telerehabilitation in MS, ensuring highly personalised and more accessible advanced treatment options to effectively manage cognitive challenges and improve quality of life.

## 5. Conclusions

This literature review presented recent findings on the feasibility and efficacy of cognitive telerehabilitation using digital platforms in MS. While the feasibility of telerehabilitation for cognition in MS has shown some positive results, with no technical barriers highlighted and adequate adherence, there was heterogeneity across studies regarding efficacy. Cognitive telerehabilitation offers PwMS the possibility to independently manage their rehabilitation in a familiar environment, implementing access to services. Moreover, the cost reduction associated with remote rehabilitation could enhance accessibility and alleviate financial burdens on patients, their families, and the national healthcare system, although several methodological limitations and heterogeneity in study designs have been identified as potential reasons for inconclusive findings. Future research should focus on addressing these challenges, including larger sample sizes, standardised outcome measures, and consideration of disease heterogeneity. Additionally, efforts should be made to optimise the intensity and duration of telerehabilitation interventions. By addressing these factors, we can enhance the validity, reliability, and generalisability of research findings, ultimately paving the way for more effective and accessible cognitive rehabilitation for individuals living with MS.

## Figures and Tables

**Table 1:** Included studies on digital telerehabilitation.

Study	Groups (N)	Study Aim	Cognitive Domains Targeted	Outcome Measures	Follow-Up Assessment	Blinding	Random Allocation of Participants	Active Control Condition
Hildebrandt et al. (2007) [53]	IG (17) CG (25)	Explore efficacy of remote cognitive training	Memory, working memory	Disability, motor, cognition, mood, fatigue, quality of life	No	Single-blinding	No	No
Vogt et al. (2009) [54]	IG intensive training (15) IG distributed training (15) CG (15)	Evaluate two different remote training schedules	Working memory	Cognition, fatigue, mood, quality of life	No	Not specified	No	No
Shatil et al. (2010) [55]	IG (59) CG (48)	Explore unprompted adherence to personalised remote cognitive training	Dependent on individual performance on neuropsychological examination	Cognition, mood, disability, fatigue	No	Not specified	No	No
Amato et al. (2014) [6]	IG (55) CG (33)	Explore efficacy of remote cognitive training	Attention	Cognition, depression, fatigue, everyday activities	6 months	Double-blinding	Yes	Yes
Charvet et al. (2015) [46]	IG (11) CG (9)	Explore feasibility of remote cognitive training	Processing speed, memory	Cognition, motor	No	Double-blinding	Yes	Yes
De Giglio et al. (2015) [56]	IG (18) CG (17)	Explore efficacy of remote cognitive training	Attention, processing speed, working memory	Cognition, fatigue, quality of life	No	Single-blinding	Yes	No
Hancock et al. (2015) [57]	IG (15) CG (15)	Explore efficacy of remote cognitive training	Processing speed, working memory	Cognition, mood, fatigue, quality of life	No	Double-blinding	Yes	Yes
Campbell et al. (2016) [58]	IG (19) CG (19)	Explore efficacy of remote cognitive training	Working memory, visuospatial memory, divided attention	Cognition, quality of life, mood, patient reported chronic illness management, self-efficacy, self-reported cognition, fatigue	4.5 months	Open-label	Yes	Yes
Pedullà et al. (2016) [59]	IG (14) CG (14)	Explore efficacy of remote cognitive training	Working memory	Cognition	6 months	Single-blinding	Yes	Yes
Charvet et al. (2017) [60]	IG (74) CG (61)	Explore efficacy of remote cognitive training	Processing speed, attention, working memory, executive functions	Cognition	No	Double-blinding	Yes	Yes
Messinis et al. (2020) [61]	IG (19) CG (17)	Explore efficacy of remote cognitive training	Attention, divided attention, verbal memory, visuospatial memory, executive functions	Cognition, fatigue, mood, quality of life	No	Single-blinding	Yes	Yes
Vilou et al. (2020) [62]	IG (23) CG (24)	Explore efficacy of remote cognitive training	Episodic memory, attention, processing speed	Cognition	No	Single-blinding	Yes	No
Blair et al. (2021) [63]	IG (15) CG (15)	Explore efficacy of remote cognitive training	Attention, working memory	Cognition, self-reported cognitive function, mood, pain, quality of life	6 months	Single-blinding	Yes	No

Abbreviations: CG, Control Group; IG, Intervention Group.

**Table 2:** Sample characteristics.

Study	Disease Type	Mean Age of Participants	Gender of Participants	Mean Education	Mean EDSS	Dropouts
Hildebrandt et al. (2007) [53]	RRMS (42)	IG: 42.4 CG: 36.5	IG: 5 M, 7 F CG: 12 M, 1 F	IG: 11.6 CG: 11.2	IG: 2.9 CG: 2.7	Not reported
Vogt et al. (2009) [54]	RRMS (36) SPMS (8) Chronic-progressive MS (1)	IG intensive training: 43.20 ± 8.80 IG distributed training: 43.40 ± 12.33 CG: 46.27 ± 10.53	IG intensive training: 4 M, 11 F IG distributed training: 6 M, 9 F CG: 5 M, 10 F	IG intensive training: 1.60 ± 0.51 IG distributed training: 1.47 ± 0.52 CG: 1.53 ± 0.52	IG intensive training: 3.23 ± 1.80 IG distributed training: 2.30 ± 1.09 CG: 3.20 ± 1.63	Not reported
Shatil et al. (2010) [55]	RRMS (107)	IG: 43.78 ± 12.15 CG: 41.35 ± 11.23	IG: 44 F, 15 M CG: 39 F, 9 M	Available only for the study completers	IG: 3.06 ± 1.95 CG: 2.66 ± 1.73	61 dropouts (37 IG, 24 CG)
Amato et al. (2014) [6]	RRMS (88)	IG: 40.1 ± 10.7 CG: 42.4 ± 12.9	IG: 44 F, 11 M CG: 25 F, 8 M	IG:13 ± 3.2 CG: 12.2 ± 3.5	IG: 2.5 ± 1.3 CG: 3.0 ± 1.7	14 dropouts
Charvet et al. (2015) [46]	RRMS (20)	IG: 38 ± 10.58 CG: 42 ± 12.53	IG: 7 F, 4 M CG: 7 F, 2 M	IG: 15.27 ± 2.57 CG: 13.88 ± 1.90	IG: 2 (median) CG: 2.5 (median)	4 participants did not meet the 50% cut-off for compliance (2 IG, 2 CG)
De Giglio et al. (2015) [56]	RRMS (35)	IG: 44.64 ± 7.63 CG: 42.99 ± 9.42	IG: 14 F, 4 M CG: 12 F, 5 M	IG: 13.94 ± 2.90 CG: 14.06 ± 3.57	IG: 3.25 (median) CG: 2 8 (median)	1 dropout CG
Hancock et al. (2015) [57]	RRMS (21) SPMS (5) PPMS (4)	IG: 50.65 ± 6.32 CG: 49.13 ± 10.09	24 F, 6 M	IG: 14.65 ± 2.06 CG: 16.33 ± 3.11	Not reported	31 dropouts (14 IG, 17 CG)
Campbell et al. (2016) [58]	RRMS (27) SPMS (11)	IG: 46.21 ± 6.59 CG: 48.53 ± 9.63	IG: 13 F, 6 M CG: 14 F, 5 M	IG: 14.05 ± 2.76 CG: 13.63 ± 2.89	IG: 4.42 ± 1.75 CG: 4.45 ± 1.77	
Pedullà et al. (2016) [59]	RRMS (17) SPMS (11)	IG: 49 ± 7.1 CG: 46.1 ± 11.2	IG: 9 F, 5 M CG: 11 F, 3 M	IG: 12.8 ± 3.1 CG: 10.7 ± 3.5	IG: 3.6 ± 1.6 CG: 4.1 ± 2.3	8 dropouts (4 IG, 4 CG)
Charvet et al. (2017) [60]	RRMS (89) SPMS (35) PPMS (7)	IG: 48 ± 13 CG: 52 ± 11	IG: 50 F, 24 M CG: 54 F, 7 M	IG: 14.82 ± 2.37 CG: 15.05 ± 2.55	IG: 3.50 (median) CG: 3.50 (median)	5 dropouts (4 IG, 1 CG)
Messinis et al. (2020) [61]	SPMS (36)	IG: 46.47 ± 4.1 CG: 45.29 ± 3.9	IG: 12 F, 7 M CG: 12 F, 5 M	IG: 13.89 ± 3.3 CG: 13.70 ± 2.5	IG: 5.5 (median range) CG: 6.0 (median range)	No dropouts
Vilou et al. (2020) [62]	RRMS (47)	IG: 33.5 (mean value interquartile) CG: 37.8 (mean value interquartile)	IG: 20 F, 3 M CG: 20 F, 3 M	Not reported	IG: 2.9 (mean value interquartile) CG: 3.5 (mean value interquartile)	No dropouts
Blair et al. (2021) [63]	RRMS (17) SPMS (12) PPMS (1)	IG: 51.07 ± 7.29 CG: 52.13 ± 8.71	IG: 12 F, 3 M CG: 9 F, 6 M	IG: 13.13 ± 1.13 CG: 13.73 ± 1.87	IG: 4.5 (median) CG: 4 (median)	8 dropouts (4 IG, 4 CG)

Abbreviations: CG, Control Group; EDSS, Expanded Disability Status Scale; IG, Intervention Group; PPMS, Primary Progressive Multiple Sclerosis; RRMS, Relapsing Remitting Multiple Sclerosis; SPMS, Secondary Progressive Multiple Sclerosis.

**Table 3:** Efficacy of digital telerehabilitation.

Study	Positive Results on NPS Measures	Positive Results on Other Measures	Negative Results on NPS Measures	Negative Results on Other Measures	Positive Results Maintained at Follow-Up	Effect Sizes for (Immediate) Positive Results
Hildebrandt et al. (2007) [53]	CVLT-II (learning trials and long delay free recall); PASAT	NHPT	CVLT-II (short-delay free recall, cued recall and long-delay cued recall); object alternation RTs and errors; alertness with and without cueing	EDSS; timed walked test; SF-12 bodily and mental scores; BDI; FSS	No follow-up assessment performed	Not reported
Vogt et al. (2009) [54]	Corsi blocks backward (only in the distributed training group); Digit span backward; two-back task omissions; PASAT; FST	FSMC; MFIS	Corsi blocks forward; digit span forward; SDMT	CES-D; FAMS	No follow-up assessment performed	Corsi block backward: η_p_^2^ = 0.08; Digit span backward: η_p_^2^ = 0.11; 2-back task omissions: η_p_^2^ = 0.06; PASAT: η_p_^2^ = 0.10; FST: η_p_^2^ = 0.14 (distributed training group). Digit span backward: η_p_^2^ = 0.11; 2-back task omissions: η_p_^2^ = 0.06; PASAT: η_p_^2^ = 0.10; FST: η_p_^2^ = 0.11 (high intensity training group)
Shatil et al. (2010) [55]	General memory; visual working memory; verbal auditory working memory (N-CPC)		Auditory (non-linguistic) working memory; awareness divided attention avoiding distractions; hand–eye coordination; inhibition naming; planning response time; shifting attention spatial perception; time estimation; visual perception; visual scanning (N-CPC)	Zung depression scale; EDSS; FSS	No follow-up assessment performed	General memory: η_p_^2^ = 0.207; Visual working memory: η_p_^2^ = 0.196; Verbal auditory working memory: η_p_^2^ = 0.191.
Amato et al. (2014) [6]	PASAT; SDMT (improvement also in the control group)	ESS (improvement also in the control group); MADRS (improvement also in the control group); VAS (improvement also in the control group)	Visual search; TMT A and B; SRT; SPART; WLG	FSS	PASAT; SDMT (improvement sustained also in the control group)	Not reported
Charvet et al. (2015) [46]	General composite cognitive score	Ecog	WAIS-IV letter number sequence; Corsi block; PASAT; SRT; BVMT-R		No follow-up assessment performed	General composite cognitive score: d = 1.11
De Giglio et al. (2015) [56]	Stroop test; SDMT	MSQoL-54 mental health composite, role limitations: emotional, emotional well-being, cognitive function, health distress	PASAT 3	MFIS; MSQoL-54 physical health composite, physical function, role limitations: physical, pain, energy, health perceptions, social function, sexual function, overall QoL, sexual function	No follow-up assessment performed	Stoop test: F^2^ = 0.210; SDMT: F^2^ = 0.177
Hancock et al. (2015) [57]	PASAT		SDMT; Stroop test; LNS; Digit backward; Raven’s APM; BVMT-R; COWAT; CPT-II; AVLT	BDI; STAI; MFIS; MSQOL-54	No follow-up assessment performed	PASAT: d = 0.90
Campbell et al. (2016) [58]	SDMT		CVLT-II; BVMT-R	EQ-5D; FAMS; PAM-13; USE-MS; HADS; MSNQ; FSS	Not significant	Not reported
Pedullà et al. (2016) [59]	SRT consistent long-term retrieval and delayed recall; SDMT; PASAT; WLG		SRT long term storage; SPART; WCST		PASAT; SDMT	Not reported
Charvet et al. (2017) [60]	General composite cognitive score		SRT; BVMT-R; PASAT; TMT; WAIS-IV letter number sequence; WAIS-IV digit span backward; DKEFS		No follow-up assessment performed	General composite cognitive score: d = 0.38
Messinis et al. (2020) [61]	SDMT, GVLT, BVMT-R	MFIS; BDI-FS; EQ-5D			No follow-up assessment performed	SDMT: g = 2.980; GVLT: g = 2.898; BVMT-R: g = 1.699
Vilou et al. (2020) [62]	GVLT; BVMT-R; TMT-A; Stroop test		SDMT; TMT-B		No follow-up assessment performed	GVLT: d = 0.6; BVMT-R: d = 0.38; TMT-A: d = 0.15; Stroop test: d = 0.32
Blair et al. (2021) [63]	DKEFS colour-word interference test; WAIS-III digit span backward	HADS-D	SDMT; PASAT; CVLT-II; BVMT-R; VSVT; WAIS-III spatial span forward and backward, WAIS-III arithmetic; WAIS-III digit span forward; WAIS-III letter-number sequence	BDI-FS; FSS, MSNQ, HADS-A; SF-36; DEX; CFQ; Brief COPE; PDQ; NPRS	HADS-D	DKEFS colour-word interference test: d = 0.27; WAIS-III digit span backward: d = 0.69

Abbreviations: APM, Advanced Progressive Matrices; AVLT, Auditory Verbal Learning Test BDI, Beck’s Depression Inventory; BDI-FS, Beck’s Depression Inventory—fast screening; BVMT-R, Brief Visuospatial Memory Test-Revised; CES-D, Centre for Epidemiologic Studies Depression Scale; CFQ, Cognitive Failures Questionnaire; COWAT, Controlled Oral Word Associations Test; CPT, Conners’ Continuous Performance Test; CVLT, California Verbal Learning Test; DEX, Dysexecutive Questionnaire; DKEFS, Delis–Kaplan Executive Function System; Ecog, Everyday cognition scale; EDSS, Expanded Disability Status Scale; EQ-5D, EuroQOL five-dimension questionnaire; ESS, Environmental Status Scale; FAMS, Functional Assessment of Multiple Sclerosis; FSS, Fatigue Severity Scale; FSMC, Fatigue Scale for Motor and Cognitive Functions; FST, Face Symbol Test; GVLT, Greek Verbal Learning Test; HADS-A, Hospital Anxiety Scale; HADS-D, Hospital Depression Scale; LNS, Letter–Number Sequencing; MADRS, Montgomery and Asberg Depression Rating Scale; MFIS, Modified Fatigue Impact Scale; MSNQ, Multiple Sclerosis Neuropsychological Questionnaire; MSQoL-54, 54-item Multiple Sclerosis Quality of Life Questionnaire; NCP-C, Neuropsychological Examination—CogniFit Personal Coach; NHPT, Nine Hole Peg Test; NPRS, Numeric Pain Rating Scale; PAM-13, Patient Activation Measure; PASAT, Paced Auditory Serial Addition Test; PDQ, Perceived Deficits Questionnaire; QoL, Quality of Life; RTs, Reaction Times; SDMT, Symbol Digit Modalities Test; SF-12, short form of the SF 36 health questionnaire; SF-36, Short Form Health Survey; SPART, 10/36 Spatial Recall Test; SRT, Selective Reminding Test; STAI, State–Trait Anxiety Inventory; TMT, Trail Making Test; USE-MS, Unidimensional Self-Efficacy scale for Multiple Sclerosis; VAS, visual analog scale; VSVT, Victoria Symptom Validity Test; WAIS, Wechsler Adult Intelligence Scale; WCST, Wisconsin Card Sorting Test; WLG, Word List Generation.

## Data Availability

No new data were created or analysed in this study. Data sharing is not applicable to this article.
